# Genetic polymorphisms of organic cation transporters 1 (OCT1) and responses to metformin therapy in individuals with type 2 diabetes mellitus: a systematic review protocol

**DOI:** 10.1186/s13643-018-0773-y

**Published:** 2018-07-25

**Authors:** Edith Pascale Mofo Mato, Magellan Guewo-Fokeng, M. Faadiel Essop, Peter Mark Oroma Owira

**Affiliations:** 1 Molecular and Clinical Pharmacology Research Laboratory, Department of Pharmacology, Discipline of Pharmaceutical Sciences, School of Health Sciences, University of KwaZulu-Natal, PO Box X5401, Durban, South Africa; 2 Laboratory of Public Health Research Biotechnology (LAPHER-Biotech), Biotechnology Centre, University of Yaounde I, PO Box 3851, Yaounde, Cameroon; 3 Laboratory of Molecular Medicine and Metabolism (LMMM), Biotechnology Centre, University of Yaounde I, PO Box 3851, Yaounde, Cameroon; 4 Cardio-Metabolic Research Group (CMRG), Department of Physiological Sciences, Stellenbosch University, Stellenbosch, South Africa

**Keywords:** Genetic polymorphisms, Glycemic response, Metformin, OCT1, Type 2 diabetes mellitus

## Abstract

**Background:**

Metformin is one of the most commonly used drugs for type 2 diabetes mellitus (T2DM). Despite its efficacy and safety, metformin is frequently associated with highly variable glycemic responses, which is hypothesized to be the result of genetic variations in its transport by organic cation transporters (OCTs). This systematic review aims to highlight and summarize the overall effects of OCT1 polymorphisms on therapeutic responses to metformin and to evaluate their potential role in terms of interethnic differences with metformin responses.

**Methods/design:**

We will systematically review observational studies reporting on the genetic association between OCT1 polymorphisms and metformin responses in T2DM patients. A comprehensive search strategy formulated with the help of a librarian will be used to search MEDLINE via PubMed, Embase, and CINAHL for relevant studies published between January 1990 and July 2017. Two review authors will independently screen titles and abstracts in duplicate, extract data, and assess the risk of bias with discrepancies resolved by discussion or arbitration of a third review author. Mined data will be grouped according to OCT1 polymorphisms, and their effects on therapeutic responses to metformin will be narratively synthesized. If sufficient numbers of homogeneous studies are scored, meta-analyses will be performed to obtain pooled effect estimates. Funnel plots analysis and Egger’s test will be used to assess publication bias. This study will be reported according to the Preferred Reporting Items for Systematic Review and Meta-Analysis (PRISMA) guidelines.

**Discussion:**

This review will summarize the genetic effects of OCT1 polymorphisms associated with variabilities in glycemic responses to metformin. The findings of this study could help to develop genetic tests that could predict a person’s response to metformin treatment and create personalized drugs with greater efficacy and safety.

**Systematic review registration:**

Registration number: PROSPERO, CRD42017079978

**Electronic supplementary material:**

The online version of this article (10.1186/s13643-018-0773-y) contains supplementary material, which is available to authorized users.

## Background

Metformin is recommended by major clinical practice guidelines as first-line oral anti-hyperglycemic drug for use as monotherapy in individuals with newly diagnosed T2DM [1]. It specifically decreases hepatic gluconeogenesis without enhancing insulin secretion, inducing weight gain, or increasing the risk of hypoglycemia [2].

The precise molecular mechanisms underlying metformin’s action are not well understood. It was initially suggested that one of the key actions of metformin is to activate AMP-activated protein kinase (AMPK) through a decrease in hepatic energetic status (increasing AMP: ADP and/or ADP/ATP concentration ratios) or through an upstream AMPK kinase (LKB1), leading to lowered transcription of gluconeogenic genes [3]. However, recent investigations in conditional AMPK knockout mice showed that metformin inhibits hepatic gluconeogenesis in an LKB1- and AMPK-independent manner via a decrease in hepatic energetic status [4, 5]. This preferential action of metformin in hepatocytes is due to the predominant expression of organic cation transporter 1 (OCT1), the main transporter responsible for hepatic uptake of metformin [6, 7].

OCT1 belongs to the solute carriers (SLC) 22A superfamily of polyspecific membrane proteins that play a central role in transportation of organic cations, anions, and zwitterions thus playing a major role in the cellular organic ions homeostasis [8, 9]. In human, OCT1 is most strongly expressed in the liver, whereas in rodents, OCT1 is also strongly expressed in the kidney and small intestine. In human and rat liver, OCT1 is located to the sinusoidal membrane of the hepatocytes [10]. The major function of OCT1 most likely is mediating the uptake of organic cations in hepatocytes as the initial step of biliary secretion [11].

Despite its widespread use, there are considerable variations in response to metformin therapy ranging from improvement in HbA1c levels (by up to 4%) to estimates of ~ 35% failure to achieve the glycemic goal (HbA1c level less than 7%) [12]. These variabilities in metformin efficacy clearly suggest the implication of individual genetic imprints [13]. Since genetic variations in some drug transporters can dramatically alter the pharmacokinetics and pharmacodynamics of many drugs, studies conducted in different population groups have suggested that OCT1 genetic polymorphisms could affect metformin responses [14, 15]. However, there is no consensus about its precise effect; both positive and negative findings have been reported [14, 16–19]. Consequently, there is need to review the existing studies linking OCT1 variants and metformin responses in order to (a) summarize the overall effects of OCT1 polymorphisms on therapeutic responses elicited by metformin intake and (b) to evaluate the potential role of such polymorphisms in terms of interethnic differences in response to metformin therapy.

### Previous literatures

Preliminary studies have reported the effects of OCT1 variants on metformin responses in T2DM individuals [16, 20–24]. In a meta-analysis carried out by Dujic et al. (2017), in order to clarify the significance of genetic variations of metformin transporter genes on glycemic response to metformin, nine candidate variants in membrane transporter genes (thereof 3 variants from OCT1) were analyzed in 7968 individuals across the cohorts of the Metformin Genetics consortium (MetGen). The authors show that the candidate variants in membrane transporter genes showed no significant effect on metformin response assessed as HbA1c reduction in patients with T2DM [14].

### Why we will conduct this systematic review?

The previous meta-analysis studied the effects of three candidate OCT1 variants on glycemic response to metformin in 7968 MetGen participants of European ancestry.

- Our study will not be restricted on pre-specified OCT1 variants. We will analyze every OCT1 variant identified that could be related to metformin response. Of note, Seitz et al. performed a global scale population analysis of OCT1 variants and identified 85 variants in 52 worldwide population groups that included sub-Saharan Africa, the Middle East and North Africa, Central Asia, East Asia and Oceania, Europe, and America [15].
- Our study will add studies that were not included in the previous meta-analysis [17, 22–26].
- Genetic variation frequencies differ among different ethnicities, which may be associated with variation of susceptibility to adverse drug reactions among the different populations [27]. In this study, we will also compare the allele frequency distribution of OCT1 genetic variants among different ethnicities.

### Objective

The objective of this systematic review is to highlight and summarize the overall effects of OCT1 polymorphisms on therapeutic responses to metformin and to evaluate their potential roles in terms of interethnic differences with metformin therapy.

## Methods

This systematic review protocol is reported following the Preferred Reporting Items for the Systematic Reviews and Meta-analysis Protocols (PRISMA-P) 2015 Checklist (Additional file 1) [28].

### Type of participants

Participants included in eligible studies must be diagnosed with T2DM and treated with metformin monotherapy for at least 3 months.

Most guidelines (American Diabetes Association, European Association for the Study of Diabetes, American Association of Clinical Endocrinologists) for the management of T2DM recommend an initial approach consisting of lifestyle changes and monotherapy, preferably with metformin. This recommendation is applicable if a patient is diagnosed with an initial HbA1c less than 9% and no existing contraindications (eGFR < 30 ml/min/1.73 m2) [29]. Three-month period on metformin monotherapy is reasonable to assess for glycemic improvement. Treatment modification is recommended when the HbA1c target (HbA1c > 7%) is not achieved or maintained by metformin monotherapy at maximal tolerated dose over 3 to 6 months [2].

### Type of exposure

We will include studies in which participants were genotyped to investigate genetic variants of OCT1.

### Comparators

The comparators are the responders and non-responders to metformin treatment. Response to metformin will be graded based on HbA1c level. Non-responders will constitute patients whose HbA1c levels declined by less than 1% after 3 months of treatment. Responders will be cases where HbA1c levels decreased by 1% or more after 3 months of treatment.

### Outcomes

The primary outcome is the clinical effect of OCT1 polymorphisms on metformin response. Genetic polymorphisms of OCT1 will include single-nucleotide polymorphisms (SNPs), deletions, duplications, and copy-number variants. Where possible, effect estimate will include odds ratio and relative risk for the genetic variant effects in responders compared to non-responders.

Secondary outcomes include effects of OCT1 polymorphisms on fasting plasma glucose (FPG) and postprandial plasma glucose (PPG) after treatment with metformin monotherapy. FPG and PPG will be compared between responders and non-responders. The incidence of gastrointestinal side-effects will be also compared between responders and non-responders where possible.

### Eligibility criteria

#### Inclusion criteria

- Cross-sectional, case-control, and cohort studies assessing the genetic effects of OCT1 variants on metformin responses (HbA1c, fasting plasma glucose levels, post-prandial glucose levels, and gastrointestinal side effects including nausea, vomiting, and diarrhea) in T2DM individuals, published between January 1990 and July 2017 without any geographical restriction
- Studies published in English or French
- Studies in which participants received metformin monotherapy as initial anti-hyperglycemic therapy for at least 3 months

#### Exclusion criteria

We will not consider:

- Letters, reviews, case reports, editorials, and comments
- Studies conducted with normal or pre-diabetes participants. Pre-diabetes is defined according to the following criteria: impaired fasting blood glucose (IFG) values between 100 and 125 mg/dL after at least 8 h of fasting, and/or glucose intolerance (ITG) when glycaemia values are between 140 and 199 mg/dL 2 h post oral administration of a 75 g glucose load (OGTT), and/or if the values of glycosylated hemoglobin (HbA1c) are between 5.7 and 6.4%
- Studies in which participants received only one dose of metformin
- Studies in which participants also have other conditions like chronic gastrointestinal diseases, chronic liver disease, cholelithiasis, chronic pancreatitis, inflammatory bowel disease, gastroduodenal ulcer, chronic kidney disease, and endocrine disorders
- Studies in which relevant data on metformin responses is lacking or impossible to extract

### Search strategy for identifying relevant studies

We will search the following electronic databases: MEDLINE via PubMed, Embase, and CINAHL from January 1990 to July 2017 without any geographical restrictions. The choice of 1990 as onset date was made on the basis that OCT1 was cloned and functionally characterized in the early–mid-1990s [30, 31]. The search strategy based on the combination of relevant terms will be designed by a librarian. The main search strategy conducted in MEDLINE via PubMed is shown in Table 1. This search strategy will be adapted for possible extension to other databases and will be updated as we progress through the review. We will also manually search reference lists from relevant studies and contact experts in the field in order to identify additional eligible studies.

**Table 1:** Search strategy in MEDLINE/PubMed

Search	Search terms
#1	“type 2 diabetes” OR Diabetes (MeSH Terms)
#2	“genetic markers” OR “genetic polymorphism” OR “Single nucleotide polymorphism” OR “Polymorphism” OR “variant” OR “gene” OR “allele” OR Genetic (MeSH Terms)
#3	“Organic Cation Transporter 1” OR “OCT 1” OR solute carrier family 22 organic cation transporter, member 1 (MeSH Terms)
#4	#1 AND #2 AND #3

### Data collection and analysis

#### Selection of studies for inclusion in the review

Two review authors (EPMM and MGF) will independently identify articles and sequentially screen their titles and abstracts for eligibility. Thereafter, full texts of articles deemed potentially eligible will be retrieved. Further, these review authors will independently assess eligibility for inclusion in the review based on the inclusion and exclusion criteria. Any disagreements between the two review authors will be resolved by consensus and arbitration of a third review author if necessary. A PRISMA (Preferred Reporting Items for Systematic Review and Meta-Analysis) flow diagram [32] will document the process of literature selection and reasons for exclusion.

### Data extraction and management

Two review authors (EPMM and MGF) will independently extract data in accordance with the methods outlined in the Cochrane Handbook for systematic reviews of interventions. A data extraction form will be designed. Approximatively 10% of the eligible studies will be randomly selected and used as pilot in the data extraction sheet in order to ensure its suitability.

Data will be collected on the first author name, year of publication, geographical location (country where the study was performed), study design, sample size, participant characteristics (mean or median age, age range, proportion of males), duration of treatment with metformin monotherapy, relevant OCT1 polymorphism, minor allelic frequencies in each population with Hardy Weinberg equilibrium if available, and primary outcome measurements (measure of metformin response after treatment with metformin). Gastrointestinal side effects, glycated hemoglobin A1 (HbA1c) levels, fasting plasma glucose (FPG), and post-prandial plasma glucose (PPG) concentrations after the treatment with metformin will be used as indices for metformin responses. Any disagreements between the two review authors will be resolved through discussion and by consulting a third author if necessary. Should any article be duplicated, we will contact the corresponding author and include the more relevant version. For managing missing data, we will contact the corresponding author of the respective studies in an attempt to obtain the required details. If no correspondence is received, the study will be included in the systematic review and discussed in the narrative summary.

### Data analysis including assessment of heterogeneity

Study characteristics and the effect estimates of OCT1 polymorphisms on metformin responses will be presented in full, in tabular form. Since this effect varies from one study to another, we will derive the pooled estimate of each polymorphism investigated in multiple studies (≥ 2), by using a random effect model.

Effects of potential confounding variables associated with metformin responses including metformin dosing, duration of treatment with metformin monotherapy, lifestyle changes, and drugs interactions will be dealt by using multivariable meta-regression analysis. All statistical analyses will be carried out using Stata statistical software version 14 (Stata Corporation, College Station, Texas, USA).

Statistical heterogeneity among the included studies will be assessed by the *X*^2^ test on Cochrane’s Q statistic. A *P* value less than 0.1 will indicate significant heterogeneity. The *I*^2^ statistic test will be further used to quantify the heterogeneity in the measure of association across studies. Values of 25%, 50%, and 75% for *I*^2^ will represent, respectively, low, medium, and high heterogeneity [33]. Where substantial heterogeneity is detected, a subgroup analysis will be performed to investigate the possible sources of heterogeneity using the following grouping variables: metformin dosing, sample size, lifestyle changes, practice of physical activity, and genotyping methods. If included studies differ significantly in design, sampling, and outcome measures, a narrative synthesis of the findings will be provided.

### Subgroup analysis

We will conduct a subgroup analysis based on metformin dosing, sample size, lifestyle changes, and the genotyping method used to detect genetic variants. For the factor metformin dosing, < 1.500 mg vs > 1.500 mg will be compared. For the sample size, small vs large will be compared. For lifestyle changes, be on diet (yes vs no) and practice of physical activity (yes vs no) will be compared. For the genotyping methods, used Taqman vs others methods will be compared. Pooled odds ratios (ORs) and 95% confidence intervals (CI) in each subgroup will be calculated. The heterogeneity between subgroups will be detected by using the *X*^2^ test on Cochrane’s Q statistic.

### Assessment of publication and reporting biases

We will assess publication bias by using standard approaches including Funnel Plots and Egger tests if enough eligible studies are available [34]. The reporting quality of each study will be independently assessed by two review authors (EPMM and MGF) using the STREGA (Strengthening the Reporting of Genetic Association Studies) statement [35] which offers guidelines for reporting of individual genetic association studies.

### Assessment of methodological and evidence qualities

Studies deemed fit for inclusion in the systematic review will be scored for methodological quality using the Newcastle-Ottawa assessment scale (NOS) [36]. Each domain will be rated with “high,” “low,” or “unclear” with regard to the risk of bias, with free text explanations.

We will also use the Grading of Recommendations Assessment, Development, and Evaluation (GRADE) methodology to assess the quality of evidence for each outcome. Quality rating of overall evidence will be downgraded according to five factors: risk of bias, inconsistency, indirectness, imprecision, and publication bias. In addition and where appropriate, the reasons to upgrade the evidence quality will include a large magnitude of effects, a dose-response gradient, and plausible residual confounding that would reduce a demonstrated effect or suggest a spurious effect when results show no effect. We will integrate downgrading and upgrading factors to obtain an overall quality of evidence for each outcome of interest. Overall quality of evidence will be then ranked as high, moderate, low, or very low as specified by the GRADE approach [37, 38].

## Discussion

Type 2 diabetes a chronic degenerative metabolic disease represents a major medical and public health problem. After lifestyle changes failure, metformin is prescribed as first-line treatment for T2DM. However, metformin is not a panacea. Clinical practice indicates that there are considerable inter-individual variations in metformin response, with about 35% of patients failing to achieve initial glycemic control on metformin monotherapy [39, 40]. Several lines of evidence based on pharmacogenetic research have demonstrated that genetic variation is one of the major factors affecting metformin responses [41]. In addition, it is well known that genetic polymorphisms in gene encoding drug-metabolism enzymes and drug transporters contribute to interindividual variabilities in the pharmacokinetics/pharmacodynamics profiles of clinical drugs [42]. The most studied transporter regarding the impact of genetic variation on metformin action has been OCT1. The gene encoding OCT1 is highly polymorphic, with a number of coding missense single nucleotide polymorphisms that affect its activity and function [43]. This study will systematically review worldwide reports that have investigated an association between any OCT1 genetic polymorphism and therapeutic response to metformin in T2DM patients. Understanding the diversity of genetic markers associated with drug response across different global populations is essential to set ethnicity-specific reference for adverse drug reactions and identify patient groups whose genetic characteristics put them at special risk from either excessive or reduced pharmacologic effects of metformin. In terms of limitations, definitive conclusions may not be possible due to the small sample size and heterogeneity of study design and protocols.

## Additional file

Additional file 1: PRISMA-P 2015 checklist. (DOC 82 kb)

## Abbreviations

AMPK: Adenosine monophosphate-activated protein kinase
FPG: Fasting plasma glucose
HbA1c: Glycosylated hemoglobin
LKB1: Liver kinase B1
OCT1: Organic cation transporter 1
OCTs: Organic cation transporters
PPG: Postprandial plasma glucose
PRISMA: Preferred Reporting Items for Systematic Review and Meta-Analysis
STREGA: Strengthening the Reporting of Genetic Association Studies
TDM: Type 2 diabetes mellitus
